# Round the Hospitals

**Published:** 1918-09-21

**Authors:** 


### ROUND THE HOSPITALS.

Probably the most important innovation in British training-schools for nurses has been the introduction of the sister-tutor. It certainly confers distinction upon the British Women's Hospital Committee that they should have enabled the College of Nursing authorities to offer College members three studentships tenable at King's College for Women (University of London) whereby each holder of a studentship would receive a year's course of study to enable her to qualify for the post of sister-tutor at an important training-school for nurses. At the first election, the entrance examination for which the advertisement described as non-competitive, there were twelve candidates, including one from Scotland. The papers were set and the answers scrutinised by the King's College for Women. The results, with the hospital records and references of the candidates, were then considered by the Council of the College of Nursing, and the awards were made to the following three ladies:—Miss Madge Elizabeth Abram, Assistant Matron, Royal Infirmary, Huddersfield; Miss Dorothy Edith

**ROUND THE HOSPITALS**—(*continued*).

Bannon, sister of the surgical ward at St. Thomas's Hospital; Miss Diana Marion Edgell, night charge nurse of the maternity ward at St. Thomas's Hospital. Miss Abram was trained at Manchester Royal Infirmary, and Miss Bannon and Miss Edgell at St. Thomas's Hospital. These three successful students will enter as residents at King's College on October 1. The consideration of these facts makes us wish we were young and fortunate enough to be one of the successful candidates. We are asked to state for the information of parents, tutors, graduates, and nurses that the whole or separate subjects of the year's course of study to qualify for the post of sister-tutor may be taken on payment of the fees, the amount of which and other particulars may be obtained from the secretary of King's College for Women (University of London), Campden Hill Road, W. 7.

We are asked to remind all members of the College of Nursing who are resident in London that the meeting for the establishment of a London Centre is to be held next Wednesday, the 25th inst., at 7 P.M., at the College of Ambulance, 3 Vere Street, Cavendish Square, W. It is hoped that members will make every effort to be present, as important matters will come up for discussion.

The new Centre of the College of Nursing, inaugurated at Cambridge on September 11, opens under very promising auspices, and is likely to be second to none in influence. Miss Crookenden, the able matron of Addenbrooke's Hospital, took the chair at the preliminary meeting, and was supported by the Rev. J. Bascombe Locke, chairman of the hospital. The audience was large and enthusiastic, and unanimously voted in favour of forming a Centre of the College at Cambridge. Mr. Bascombe Locke told the nurses they would probably soon secure State Registration by a united effort, since the granting of the Parliamentary franchise to women had largely increased their power to influence the event. Miss Cowlin, the organising secretary of the College, spoke forcibly and well, urging that the Cambridge Centre, being in touch with the leaders of higher education for women, ought to exert all its forces on behalf of the higher education of nurses. A further meeting of members has been fixed for September 25, when the honorary officers and committee will be appointed for the remainder of the College year.

The post-graduate course for nurses during the winter session, arranged to take place at 6 P.M. in the lecture-room of the Nurses' Home, Royal Infirmary, Leicester, affords a good example of the advantage to nurses of establishing a local Centre of the College of Nursing. The first lecture, on September 24, will be delivered by Colonel Bond, C.M.G., F.R.C.S., A.M.S., on "The Treatment of War Wounds." On October 22 and November 19 Colonel Astley V. Clarke, M.D., T.F. (R.), will lecture on "Shell-shock and Allied Conditions"; December 17, Dr. C. Killick Millard, M.D., D.Sc., M.O.H., "Infectious Diseases"; January 21, Dr. Bessie Symington, M.B., B.S. Lond., on "Venereal Diseases"; February 25, Dr. E. C. Hadley, M.D., B.S. Lond., "The Application of Physics in Nursing"; March 25, Major J. D. Slight, M.A., M.D., R.A.M.C. (T.), "Some Remarks on Heart Disease." The lectures are open to all nurses, College members and others. Season tickets 5s., single lecture 1s.

At an Investiture held in the quadrangle of Buckingham Palace on September 11 the King personally conferred the decoration of the Royal Red Cross, First Class, on the following:—*Q.A.I.M.N.S.*—Matron Kate Roscoe, Sister Helen Day. *T.F.N.S.*—Matron Sarah Cockrell and Sister Joseph Cardozo. The following received the honour of being admitted Associates of the Royal Red Cross:—*Q.A.I.M.N.S.*—Sister Dora Grayson. *Q.A.I.M.N.S.R.*—Sisters Lily Jenkins and Mary Wedderspoon. *Q.A.I.M.N.S. India.*—Matron Marion Knapp. *T.F.N.S.*—Sister Annie Gibbins. *Civil Nursing Service.*—Matron Annie Rastall, Assistant Matrons Edith Cockeram, Jean Dumble. *B.R.C.S.*—Sisters Lily Griffiths and Ruth Nicholas. *Canadian A.N.S.*—Sister Gwendolen Spalding. *V.A.D.*—Misses Bertha Cattell, Maude Epps, and Elizabeth Sinclair-White. Sister Winifred Hawkins, T.F.N.S., received the Military Medal for bravery under enemy fire. The King has awarded the Royal Red Cross, Second Class, to Miss Letitia Reeves, Australian Auxiliary Hospital, Welwyn, for valuable nursing services in connection with the war.

It is matter for general congratulation that provincial hospitals giving four years' training are beginning to follow the lead given by some of the major training-schools in London and are offering three years' training to V.A.D. members who have served for two years during the war in a military hospital. The Liverpool Royal Infirmary was, we believe, the first to make definite arrangements for the general training on a three years' agreement of a limited number of V.A.D. probationers desirous of becoming fully-trained nurses. This privilege has now been granted by King Edward VII.'s Hospital, Cardiff, which is thus second in the field, and we confidently expect that many other well-known institutions will follow suit.

It is honourable to Manchester and Salford that in spite of the unexampled demands on the hospitals to furnish nurses for war work during the last four years, the nursing staffs of the various hospitals have never been suffered to fall below requirements. The number of candidates for training, if less ample than formerly, has not failed to afford a good choice of women suitable for probationers. We learn, however, that, in common with other parts of the country, Manchester hospitals are suffering badly for lack of ward-maids. At St. Mary's Hospital, Busholme, for instance, there are but six maids in place of twenty-two. The demand for unskilled female

**ROUND THE HOSPITALS—*(continued)*.**

labour has never been so great in the history of this country, and the wages never so high. It seems that hospitals must modify the working conditions and raise the wages of their maids if they are successfully to compete with other organisations.

THE press is being largely invoked to make known to the public the need for more probationers in various kinds of hospitals. The worst shortage is in fever and tuberculosis institutions, which is very natural, as the outside public has still a considerable dread of infection and but little idea of the advantageous conditions under which the nursing of tuberculosis and fever patients is carried out. The first condition of success in attracting probationers to the Service is to remove this dread founded on ignorance, and far too little is being done in this direction. The Labour Exchanges, however successful in recruiting for other branches of national work, do not seem to press the needs and advantages of the Municipal Nursing Service with conspicuous ability. Generally speaking, it is the sight of nurses at work which inspires the wish to come and do likewise. If the M.A.B. institutions could be rendered a little less inaccessible to the public there would be more candidates.

THE King paid a visit while in France to the hospital where four men of the American Friends' Ambulance who had been injured in the recent air raid on their headquarters were recovering. Part of the building in which these men were working was blown away altogether. One man owed his life to the fact that the concussion blew his bed across the room a moment before the cupola of the building fell across where he had been sleeping. The Friends' Unit is now moving into other quarters, but they are not likely to secure very quiet times, as the house next door has been hit from the sea, from the sky, and by long-range gunfire.

THE new hostel in Holland Park for members of the Queen Mary's Army Auxiliary Corps has now been provided with an indispensable sick-bay, which has been opened at 53 Holland Park for the London district. The Lady Superintendent is Miss R. MacEwen; there is a resident medical officer, and nursing members of the corps minister to the patients. There is room for fifty patients in need of care for slight ailments or after-hospital treatment, or simply tired out. The upper floor provides isolation for mild infectious cases. It is very satisfactory that the principle of treating workers at the outset of ill-health, instead of, as formerly, ignoring symptoms till serious illness set in, has now been fully established in all the Services. It is a principle which has reduced the sickness rate among nurses in well-ordered hospitals to a vanishing figure.

THE trial of Eva Grace Thompson, night nurse at the Infant Welfare Centre, Lower Sydenham, charged with the murder of a baby under her care, took place on September 13 at the Central Criminal Court, before Mr. Justice Darling. The jury found her guilty, but that she was insane at the time, and she was ordered "to be detained until His Majesty's pleasure be known," or, in other words, imprisoned as a criminal lunatic. There had been a long history of drug-taking and irresponsible conduct, and it is clear that she ought not to have been engaged merely on the written recommendation of "a lady who knew her and her work," for nurses who can offer no professional testimonials are self-convicted of incompetence or worse.

THE case illustrates the extreme importance of introducing some inspection and control among the very extensive body of untrained or partly-trained women who do nursing work. Without certificates, depending on chance recommendations, this degraded woman was able to secure a post in which ten or eleven infants were left under her care night after night for nearly twelve hours. Two children died from injuries she inflicted, and a third was injured but recovered. Ought not the women employed for helpless invalids and infants to be compelled by law to show a licence? We have frequently had to note the ease with which unsuitable women, calling themselves nurses, procure posts in which almost unlimited powers for doing harm are put within their reach. The State Registration of fully-trained nurses will leave the public as helpless as ever in this respect unless order be introduced among the heterogeneous company of the nurse-attendants.

THE contrast lately brought to notice through the Report of the Mansion House Council between the nursing of children in voluntary hospitals and their treatment in Poor-Law infirmaries ought not to pass unnoticed. In the voluntary children's institutions the average number of beds to a nurse varies from 2½ to 4. In Poor-Law infirmaries the average is from 8 to 10 beds. If it be asked why institutions mostly in chronic want of money employ so many more nurses in their children's wards than municipal authorities, there can be but one answer. The hospital employs the minimum of nurses which can give proper care to the little patients. Below that minimum the children's wants, some of their wants, must perforce go unsupplied. Perhaps the features which strike most painfully the visitor to Poor-Law sick children are the silence, the want of toys, the absence of any attempt to cheer the little sufferers. There is no doubt that the gaiety and beauty of the children's wards in hospitals have their definite curative function. A children's ward without music, paintings, or toys is but half-equipped. Of what are the children thinking all day long when no one has a minute to speak to them? Of what but their own ailments, the forebodings imparted by indiscreet relatives, the ache of home-sickness? Nothing is more inexplicable than London's neglect of the children's wards in the infirmaries. It may be said

**ROUND THE HOSPITALS—(*continued*).**

of the matrons of these institutions that they perform miracles with the scanty staff allowed them. The help they get from the outside public is nil.

---

FEMALE medical and surgical progress in India has always been very closely linked with the spread of trained nursing in that country, and the admirable report of the Countess of Dufferin's Fund for Supplying Female Medical Aid to the Women of India gives important details of the work accomplished in this direction. It is provided with a fine map, is most carefully edited, and forms a valuable reference book on social conditions in India. The demand for trained native nurses is enormous and increasing, while there is a grave shortage of sisters in nearly all the hospitals capable of giving efficient training. The amount of suffering unrelieved, the depth of the ignorance and superstition which prevails, are appalling. Money and energy are urgently needed. But it is the work already accomplished, work of a vital and fruit-bearing quality, which astonishes. The movement for giving elementary instruction to the native *dai* or midwife is one of the most hopeful yet inaugurated for the help of the Indian women.

---

MUCH disappointment is caused in the training of native women as nurses or midwives by failure to realise the inevitably large proportion who have to be weeded out in the process. Even in this country the proportion of candidates unsuitable for training often amounts to 30 or 40 per cent. Hence, when we read in reports of Indian hospitals again and again that out of three or four candidates two are utterly untrainable, and another cannot submit to the life, but left after a short trial, these facts ought not to induce doubts about the possibility of training good native nurses. They show, however, that some form of preliminary discipline is exceedingly desirable. A succession of out-and-out failures in the wards upsets everything, and the overworked sisters ought to be guarded from it. In many Indian Centres the Christian missions answer the purpose of weeding out the fit from the unfit, and on all hands it is acknowledged that the best probationers are those they send on.

---

SOME feeling has been caused in nursing circles in Melbourne by the announcement of the Hospital Attendants' Board that women employees in hospitals are to receive an increase of pay varying between 2s. 6d. and 5s. a week. Nurses have no union and no Wages Board and are not affected by this decision. Whether it would be for the advantage of the profession for nurses to place themselves on a level with manual workers by forming a trade union is a matter on which opinions differ. But it is certain that should a widespread feeling of discontent, produced by inadequate pay, be found to exist among nurses the authorities cannot lightly afford to disregard it. Compared with those in this country, however, nurses' salaries in Australia are good.

---

THE remonstrance of a doctor, in a recent number of the *Westminster Gazette*, over the treatment of the ward-maids in a London military hospital forms a forcible commentary on the causes of the ward-maid shortage now so painfully apparent. Working seventy-nine hours a week (twelve hours on six days and seven on Sundays), these women, many of whom are educated, receive 23s. a week, without food, lodging, or clothing, and often have to pay tram fares from their homes. No war bonus has been given, no holidays nor half-days. There is apparently no prospect of betterment before them, and the work is of the hard and monotonous kind, which, unless it leads somewhere, is apt to have a depressing effect. There is room here for a good organiser. There should be a graded service, with promotion to higher pay after six months' service, shorter hours, and less onerous conditions. A serviceable uniform should be provided, and the service of members ought to count when they leave so that they could join another institution without losing their grade. There is a very large, though not unlimited, supply of this kind of labour. But it cannot be secured without going half-way to meet just demands.

---

TRAINED laboratory assistants and dispensers holding the Pharmaceutical or the Apothecaries' Hall certificate are much wanted by the hospital authorities in Salonika. The rates of pay are 39s. 6d. per week, head dispensers 49s. 6d., with a deduction of 14s. for board and lodging. Applications are being received by the V.A.D. Department, 18 Devonshire House, Piccadilly, W. 1.

---

THE number of entries for our Leaflet Competition already ensures its success. Competitors are asked to observe the following conditions:—(1) The leaflets should be short, from 1,000 to 2,000 words each. (2) Each leaflet should deal with one branch, and one only, of nursing work, as, for instance, children's nursing, mental nursing, public health work, etc., but competitors may send in one or more leaflets. (3) Leaflets should be written for outsiders, and explain in easy language the way to qualify for the work, the pay and prospects of workers, and the higher aspects of the work undertaken. (4) Leaflets (which should be written on one side of the paper) must reach the Editor of THE HOSPITAL, 28 Southampton St., Strand, London, W.C. 2, **not later than Saturday, September 28, 1918.** No entrance fee or form is required. Provided that thirty leaflets, at least, are received three prizes will be given, of £3 3s., £2 2s., and £1 1s. It is hoped that all who have expressed their wish to enter for the Competition will compete for a prize by sending in their leaflet in good time.

**For "The Nurse and her Newspaper," by "Ierne," see p. 538; "Mothers, Babies, and the Little Bill," p. 544; Matrons' and Sisters' Appointments, p. iv.**

